# Fibrosis-Related microRNAs in Crohn’s Disease with Fibrostenosis and Inflammatory Stenosis

**DOI:** 10.3390/ijms25168826

**Published:** 2024-08-13

**Authors:** Miha Jerala, Tinkara Remic, Nina Hauptman, Nina Zidar

**Affiliations:** Institute of Pathology, Faculty of Medicine, University of Ljubljana, Korytkova 2, 1000 Ljubljana, Slovenia; miha.jerala@mf.uni-lj.si (M.J.); tinkara.remic@mf.uni-lj.si (T.R.); nina.hauptman@mf.uni-lj.si (N.H.)

**Keywords:** Crohn’s disease, fibrostenosis, inflammatory stenosis, fibrosis, miRNA

## Abstract

Crohn’s disease (CD) is frequently complicated by strictures that can be either inflammatory or fibrostenotic. This distinction is important for deciding the best treatment course, but it can be difficult to determine clinically, sometimes even by advanced imaging techniques. We performed miRNA PCR panel screening on pooled samples of ileum with CD fibrostenosis or inflammatory stenosis. Eight miRNAs with profibrotic (*miR-93-5p*, *miR-376c-3p* and *miR-424-5p*), or fibroprotective (*miR-133a-3p*, *miR-133b*, *miR-193a-5p*, *miR-335-5p* and *miR-378a-3p*) functions described in the literature were selected for validation on 20 samples each of CD with fibrostenosis or inflammatory stenosis, with a separate sampling of the submucosa and subserosa. The results showed significant differences between the groups in subserosal samples, with upregulation of profibrotic miRNAs and downregulation of fibroprotective miRNAs in fibrostenosis compared to inflammatory stenosis. Only *miR-424-5p* showed a significant difference in the submucosa. There were significant differences in miRNA expression between subserosa and submucosa. Our results provide further evidence that the major differences between fibrostenosis and inflammatory stenosis are located in the subserosa, which is inaccessible to endoscopic sampling, highlighting the need for cross-sectional imaging or serological markers. We identify several miRNAs previously not connected to fibrosis in CD, which could potentially serve as biomarkers of fibrostenosis.

## 1. Introduction

Stenosis or stricture formation with obstructive symptoms is a frequent complication of Crohn’s disease (CD) [1]. While most stenotic strictures in CD contain a mixture of inflammation and fibrosis, they are generally divided into inflammatory stenosis and fibrostenosis based on the predominant component [2]. Inflammatory stenosis is characterized by inflammatory cell infiltration and edema of the bowel wall, while fibrostenosis is characterized by fibrosis with myofibroblast proliferation and extracellular matrix deposition [2,3,4,5]. The distinction is clinically important as inflammatory stenoses show good responses to anti-inflammatory therapies with resolution of symptoms, as well as endoscopic and histologic remission, while fibrostenosis does not respond to anti-inflammatory therapy and frequently warrants surgical treatment or endoscopic dilation [6]. Despite extensive research in fibrogenesis and inflammation, there are still many unknowns [7]. Namely, we do not know why some patients develop fibrosis and we cannot predict which patients are at risk of developing fibrostenosis or inflammatory stenosis [3].

Because stenosis is a process that involves the whole thickness of the bowel wall it cannot be reliably classified based on endoscopic examination with endoscopic biopsies. The gold standard for classifying fibrostenosis versus inflammatory stenosis is histological evaluation of the whole bowel wall after surgical resection [8]. Intestinal ultrasound (IUS) and magnetic resonance enterography (MRE) offer methods of non-invasive assessment of stenosis. However, IUS has been evaluated as inaccurate, and MRE, while more accurate, is time consuming and resource intensive and requires the application of gadolinium contrast [5,8].

There have been many studies evaluating miRNA in inflammatory bowel diseases (IBD), mostly concerning disease activity or differentiating CD and ulcerative colitis (UC), with some studies also looking at fibrosis in CD [9,10,11,12,13]. There have also been studies on the role of miRNAs in the progression to IBD-associated dysplasia and cancer [14,15], as well as miRNAs with roles both in dysplasia and fibrosis [16,17]. However, the differences between CD stenoses that are fibrotic versus inflammatory are still poorly defined. Our study aimed to identify miRNAs that are differentially expressed in fibrostenosis and inflammatory stenosis using miRNA PCR panels. We examined the submucosal and subserosal layers separately, as both represent the two focuses of fibrosis in the bowel with important differences between them. A selection of miRNAs that showed the highest difference and have a role in fibrosis based on a literature review were further validated.

## 2. Results

### 2.1. Identification of Potential miRNA Targets miRnome Panel Involved in Fibrosis

**Figure 1:**
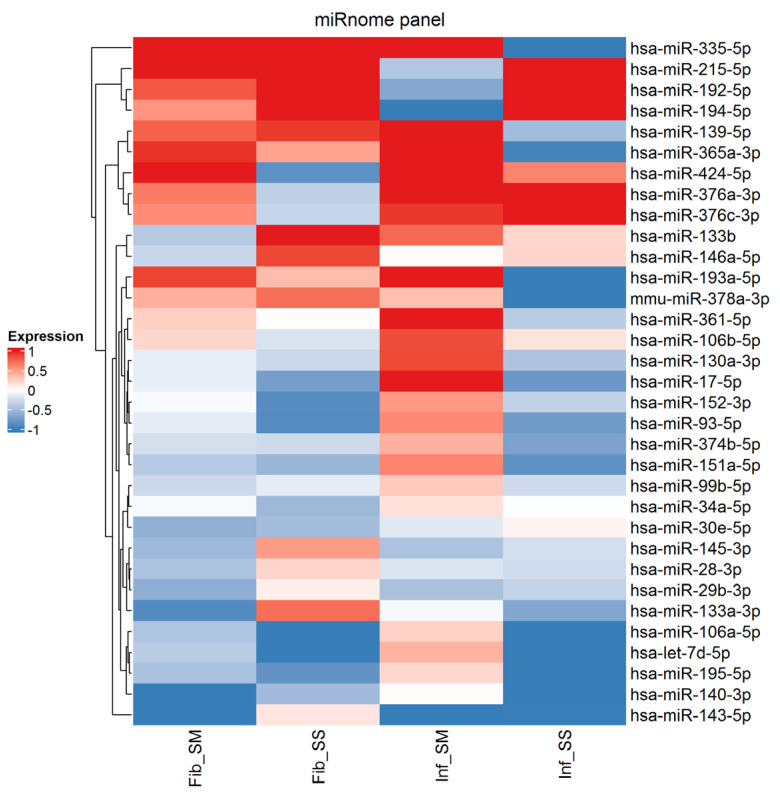
Heatmap of expression levels of miRNAs with dysregulated expression between inflammatory stenosis submucosa (Inf_SM), inflammatory stenosis subserosa (Inf_SS), fibrostenosis submucosa (Fib_SM) and fibrostenosis subserosa (Fib_SS). The red color indicates higher expression levels and the blue color indicates lower miRNA expression levels.

We examined potential targets, which had dysregulated expression (dysregulation > 2) between either subserosal and submucosal layers within the same diagnosis (i.e., Inf_SM and Inf_SS) or between different diagnoses in the same layer (i.e., Inf_SM and Fib_SM) (Figure 1). Specifically, we selected eight target miRNAs, which were previously reported to have profibrotic effects (*miR-93-5p* [18,19,20,21,22,23,24], *miR-376c-3p* [25,26] and *miR-424-5p* [27,28,29,30]) or fibroprotective effects (*miR-133a-3p* [16,17,31,32], *miR-133b* [33,34], *miR-193a-5p* [35], *miR-335-5p* [36,37] and *miR-378a-3p* [38,39,40]). The expression data for all miRNAs included in the panel can be found in Supplementary Table S1.

### 2.2. Expression of miRNA with Profibrotic Effects

To validate the results of the miRNome panels for the selected miRNAs with previously reported profibrotic effects (*miR-93-5p*, *miR-376c-3p* and *miR-424-5p*), we performed qPCR for individual samples of each study cohort.

**Figure 2:**
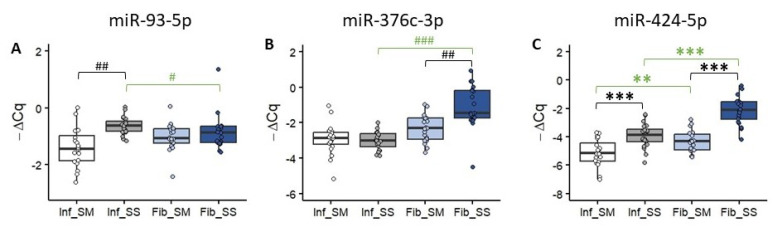
Expression of miRNA with profibrotic effects in Crohn’s disease with inflammatory stenosis (Inf) or fibrostenosis (Fib). (**A**) Box-plot with individual −ΔCq value representation of *hsa-miR-93-5p* expression in Inf_SM (white), Inf_SS (grey), Fib_SM (light blue) and Fib_SM (dark blue). (**B**) Box-plot with individual −ΔCq value representation of *hsa-miR-376c-3p* expression in Inf_SM (white), Inf_SS (grey), Fib_SM (light blue) and Fib_SM (dark blue). (**C**) Box-plot with individual −ΔCq value representation of *hsa-miR-424-5p* expression in Inf_SM (white), Inf_SS (grey), Fib_SM (light blue) and Fib_SM (dark blue). Statistical analysis: Black asterisks represent significant differences calculated using the paired sample *t*-test (***—*p* < 0.001). The green asterisks represent significant differences calculated using the independent sample *t*-test (**—*p* < 0.01, ***—*p* < 0.001). Black hash marks represent significant differences calculated using the Wilcoxon signed-rank test (##—*p* < 0.01). The green hash marks represent significant differences calculated using the Mann–Whitney U test (#—*p* < 0.05, ###—*p* < 0.001). Legend: Fib—fibrostenosis, Inf—inflammatory stenosis, SM—submucosal layer, SS—subserosal layer.

*miR-93-5p* was significantly upregulated in the subserosal layer compared to the submucosal layer of only Inf CD (*p* < 0.01), while there were no significant differences between the submucosal and subserosal layers in Fib CD (Figure 2A). There was also a significant upregulation in the subserosal layer of Inf CD compared to the subserosal layer of Fib CD (*p* < 0.05, Figure 2A).

*miR-376c-3p* was significantly upregulated in the subserosal layer compared to the submucosal layer only in Fib CD (*p* < 0.01), while there were no significant differences between the submucosal and subserosal layers in Inf CD (Figure 2B). There was also a significant upregulation in the subserosal layer of Fib CD compared to the subserosal layer of Inf CD (*p* < 0.001, Figure 2B).

Lastly, *miR-424-5p* was significantly upregulated in the subserosal layer compared to the submucosal layer in both Inf (*p* < 0.001) and Fib CD (*p* < 0.001, Figure 2C). There was also a significant upregulation in the subserosal layer of Fib CD compared to the subserosal layer of Inf CD (*p* < 0.001, Figure 2C). Moreover, there was a significant upregulation in the submucosal layer of Fib CD compared to the submucosal layer of Inf CD (*p* < 0.01, Figure 2C).

### 2.3. Expression of miRNA with Fibroprotective Effects

To validate the results of the miRNome panels for the selected miRNAs with previously reported fibroprotective effects (*miR-133a-3p*, *miR-133b*, *miR-193a-5p*, *miR-335-5p* and *miR-378a-3p*), we performed qPCR for individual samples of each study cohort. 

**Figure 3:**
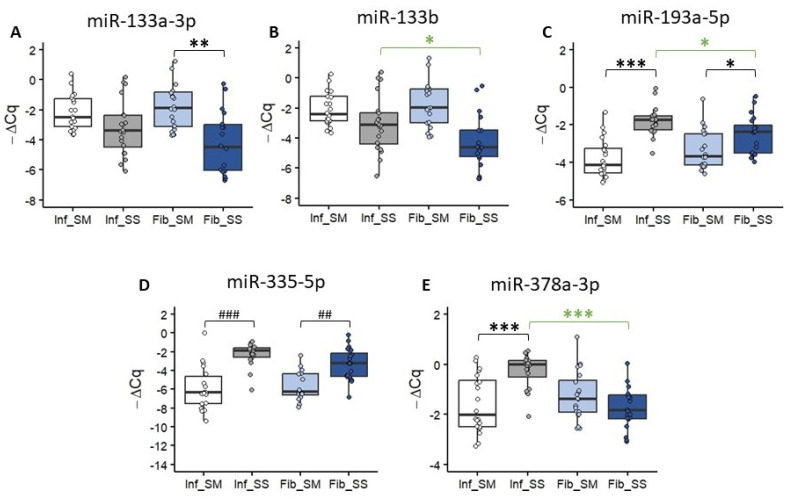
Expression of miRNAs with fibroprotective effects in Crohn’s disease with inflammatory stenosis (Inf) or fibrostenosis (Fib). (**A**) Box-plot with individual −ΔCq value representation of *hsa-miR-133a-3p* expression in Inf_SM (white), Inf_SS (grey), Fib_SM (light blue) and Fib_SM (dark blue). (**B**) Box-plot with individual −ΔCq value representation of *hsa-miR-133b* expression in Inf_SM (white), Inf_SS (grey), Fib_SM (light blue) and Fib_SM (dark blue). (**C**) Box-plot with individual −ΔCq value representation of *hsa-miR-193a-5p* expression in Inf_SM (white), Inf_SS (grey), Fib_SM (light blue) and Fib_SM (dark blue). (**D**) Box-plot with individual −ΔCq value representation of *hsa-miR-335-5p* expression in Inf_SM (white), Inf_SS (grey), Fib_SM (light blue) and Fib_SM (dark blue). (**E**) Box-plot with individual −ΔCq value representation of *hsa-miR-378a-3p* expression in Inf_SM (white), Inf_SS (grey), Fib_SM (light blue) and Fib_SM (dark blue). Statistical analysis: Black asterisks represent significant differences calculated using the paired sample *t*-test (*—*p* < 0.05, **—*p* < 0.01, ***—*p* < 0.001). The green asterisks represent significant differences calculated using the independent sample *t*-test (*—*p* < 0.05, ***—*p* < 0.001). Black hash marks represent significant differences calculated using the Wilcoxon signed-rank test (##—*p* < 0.01, ###—*p* < 0.001). Legend: Fib—fibrostenosis, Inf—inflammatory stenosis, SM—submucosal layer, SS—subserosal layer.

*miR-133a-3p* was significantly upregulated in the submucosal layer compared to the subserosal layer of only Fib CD (*p* < 0.001), while there were no significant differences between the submucosal and subserosal layers in Inf CD (Figure 3A). There were also no significant differences in either the submucosal or subserosal layers between the Inf and Fib CD (Figure 3A).

*miR-133b* was significantly upregulated in the submucosal layer compared to the subserosal layer of only Fib CD (*p* < 0.001), while there were no significant differences between the submucosal and subserosal layers in Inf CD (Figure 3B). There was also a significant upregulation in the subserosal layer of Inf CD compared to the subserosal layer of Fib CD (*p* < 0.05, Figure 3B).

*miR-193a-5p* was significantly upregulated in the subserosal layer compared to the submucosal layer of both Inf (*p* < 0.001) and Fib CD (*p* < 0.05, Figure 3C). Additionally, there was a significant upregulation in the subserosal layer of Inf CD compared to the subserosal layer of Fib CD (*p* < 0.05, Figure 3C). 

*miR-335-5p* was significantly upregulated in the subserosal layer compared to the submucosal layer of both Inf (*p* < 0.001) and Fib CD (*p* < 0.01, Figure 3D). However, there were no significant differences in either the submucosal or subserosal layers between the Inf and Fib CD (Figure 3D).

Lastly, *miR-378a-3p* was significantly upregulated in the subserosal layer compared to the submucosal layer of only Inf CD (*p* < 0.001), while there were no significant differences between the submucosal and subserosal layers in Fib CD (Figure 3E). There was also a significant upregulation in the subserosal layer of Inf CD compared to the subserosal layer of Fib CD (*p* < 0.001, Figure 3E).

## 3. Discussion

Differentiating fibrostenosis from inflammatory stenosis remains an important clinical problem with therapeutic implications [6]. In this study, we analyzed miRNAs in the submucosa and subserosa of CD with fibrostenosis and inflammatory stenosis. We divided the studied miRNAs into profibrotic and fibroprotective miRNAs, based on previous reports in the literature. Comparing the fibrostenosis and inflammatory stenosis groups, two profibrotic miRNAs, *miR-376c-3p* [25,26] and *miR-424-5p* [27,28,29,30], were significantly upregulated in subserosa in fibrostenosis compared to subserosa in inflammatory stenosis. *miR-93-5p* was first designated as profibrotic, but a more detailed review revealed a mixture of studies describing profibrotic and fibroprotective functions [18,19,20,21,22,23,24]. Since fibrosis is an intricate process with many different molecular pathways involved and miRNAs can regulate many different genes, some crosstalk is expected. In our study, *miR-93-5p* was slightly downregulated in subserosa in fibrostenosis compared to inflammatory stenosis in line with a more fibroprotective effect. The five miRNAs designated as fibroprotective, (*miR-133a-3p* [16,17,31,32], *miR-133b* [33,34], *miR-193a-5p* [35], *miR-335-5p* [36,37] and *miR-378a-3p* [38,39,40]), were downregulated in the subserosa in fibrostenosis, compared to inflammatory stenosis. Three of these, *miR-193a-5p*, *miR-378a-3p* and *miR-133b*, reached statistical significance. These findings are in line with the fibroprotective function of these miRNAs.

Strikingly, the observed differences between the miRNAs in fibrostenosis and inflammatory stenosis were mostly in the subserosal compartment, with only *miR-424-5p* showing a statistically significant difference between the submucosa of the two groups. This is in line with our previous observations that the subserosa is highly involved in fibrosis in CD, with significant differences from submucosal fibrosis [41]. We also observed that most miRNAs not only show comparable ΔCq in the submucosa of both groups but that many show significant differences in expression between the submucosa and subserosa of the same group (fibrostenosis and inflammatory stenosis). This can also be seen on the heatmap, further highlighting the differences in fibrosis between the two compartments.

The finding that most differences between fibrostenosis and inflammatory stenosis occur in the deep subserosal layer indicates that searching for a possible marker to distinguish between fibrostenosis and inflammatory stenosis using endoscopic biopsies, which sample only mucosa and submucosa, remains very challenging. On the other hand, it highlights the value of cross-sectional imaging, which provides noninvasive information on fibrosis in the deeper layers. There have also been studies examining miRNAs as serum biomarkers of fibrosis, including *miR-93-5p* [23] whose serum level was inversely correlated with recurrence at one year after surgery for fibrotic obstruction in CD. While this study does not offer promising targets for evaluation in endoscopic biopsies, several of the miRNAs that showed strong differences in the subserosa could potentially be further studied to assess their use as serum biomarkers.

Some of the miRNAs in our study have previously been studied in conjunction with IBD or CD. *miR-424-5p* was described to be upregulated in CD and UC compared to normal mucosa or IBD mimics, without mention of associated fibrosis or stenosis [42]. While Jin et al. [43] showed that *miR-133a-3p* is downregulated in DSS-induced colitis in mice, and a *miR-133a* mimic alleviates the colitis, it also has a role in IBD-associated dysplasia and progression to carcinoma [15]. *miR-133a-3p* did not show differences between the fibrostenosis and inflammatory stenosis groups in our study, its role in IBD could be more in regulating inflammation than fibrosis.

Future research should focus on the utility of the studied miRNAs as serum biomarkers of fibrosis, particularly *miR-376c-3p* and *miR-424-5p*, which were strongly upregulated in fibrostenosis. While *miR-424-5p* was also statistically significantly upregulated in the submucosa, the difference was small and likely not applicable as a marker in endoscopic biopsies. We also suggest more focus should be given to the role of the subserosal layer in fibrosis and fibrostenosis in IBD, which has been somewhat neglected compared to the more accessible mucosa and submucosa.

Our study has some limitations as it is a purely observational and retrospective study done only on archival tissue samples. Most patients received some form of medical therapy before the resection, so we cannot exclude a possible interference with previous treatment. Ideally, patients’ serum could also be tested for correlation with the most promising miRNAs, but it cannot be obtained retrospectively.

In conclusion, the analysis of miRNAs between CD with fibrostenosis and inflammatory stenosis revealed significant differences in miRNA expression, with upregulation of profibrotic and downregulation of fibroprotective miRNAs in the fibrostenotic groups. The differences, however, were mostly observed in the subserosal layers, with only *miR-424-5p* showing a significant difference in the submucosal layers, highlighting the role of the subserosa in fibrosis and the need for techniques that evaluate the whole bowel wall or perhaps serum miRNAs.

## 4. Materials and Methods

### 4.1. Patient Cohorts and Sample Selection

Samples of small intestine and ileocecal resections performed because of stenotic obstruction due to CD between 2019 and 2023 were retrieved from the archive of the Institute of Pathology, Faculty of Medicine, University of Ljubljana. The original hematoxylin and eosin slides from areas of stenosis were reviewed and assessed as fibrostenosis (stenosis with severe fibrosis and mild to moderate inflammation) or inflammatory stenosis (stenosis with no or mild fibrosis and severe inflammation). Consecutive samples from 20 patients with fibrostenosis and 20 patients with inflammatory stenosis, selected from areas of stenosis, were included in the study. The patients’ major demographic data are summarized in Table 1.

### 4.2. Ethics

This study was approved by the National Medical Ethics Committee of the Republic of Slovenia (No. 0120-139/2019/4) and performed in accordance with the guidelines of the Declaration of Helsinki.

### 4.3. Isolation of Total RNA

Representative samples (n = 40) with well-characterized submucosal (SM) and subserosal (SS) layers were selected to compare the expression of miRNA between patients with CD with fibrostenosis (Fib; n = 20) and patients with CD with inflammatory stenosis (Inf; n = 20) in the SM and SS of the ileum. For each layer, 8–10 punches with a 0.6-diameter needle were taken from tissue blocks. 

Total RNA was isolated using the MagMax FFPE DNA/RNA Ultra kit (A31881, Applied Biosystems, Thermo Fisher Scientific, Waltham, MA, USA) according to the manufacturer instructions with adjusted protease digestion parameters to an overnight incubation at 55 °C with 15s shaking at 300 rpm every 4 min. The RNA quality was assessed using a NanoDrop 1000 spectrophotometer (Thermo Fisher Scientific, Waltham, MA, USA) while the RNA concentration was determined using a Qubit Fluorimetric Quantification (Thermo Fisher Scientific, Waltham, MA, USA) following their respective manufacturer instructions. Isolated total RNA was stored at −80 °C. 

### 4.4. miRNA Expression Profiling

To identify potentially differentially expressed miRNA targets between CD with Fib and Inf in different layers of the ileum, we used the miRNome PCR Panels I + II, V5 (YAHS-312Y, Qiagen GmbH, Hilden, Germany). 

### 4.5. First-Strand cDNA Synthesis

Firstly, two sample pools per study cohort were prepared, each containing five samples, representing the following cohorts: Fib_SM_1, Fib_SM_2, Fib_SS_1, Fib_SS_2, Inf_SM_1, Inf_SM_2, Inf_SS_1 and Inf_SS_2. First-strand cDNA synthesis was performed with the miRCury LNA RT kit (339340, Qiagen GmbH, Hilden, Germany) according to the manufacturer’s instructions. Briefly, a 40 μL reverse-transcription reaction contained 8 μL of total RNA (5 ng/μL), 2 μL of UniSp6 spike-ins, 4 μL of 10× miRCURY RT Enzyme Mix, 8 μL of 5× miRCURY RT Reaction Buffer and 18 μL of RNase-free water. All reaction mixtures were incubated for 60 min at 42 °C and 5 min at 95 °C. If not used immediately, the synthesized cDNA was stored at −20 °C. 

### 4.6. Quantitative Real-Time PCR (qPCR)

The miRNome PCR Panels I + II, V5 (YAHS-312Y, Qiagen GmbH, Hilden, Germany) were used to examine the expression of 752 different miRNA. The qPCR reaction mix was prepared and the reaction was performed according to the manufacturer’s instructions. Briefly, an 8000 μL qPCR reaction mix contained 40 μL of undiluted cDNA template, 40 μL of 200× ROX Reference Dye, 4000 μL of 2× miRCURY SYBR Green Master Mix and 3960 μL of RNase-free water. After thorough mixing, 10 μL of the reaction mix was dispensed into each well of the two miRNome PCR panels. Finally, the qPCR reactions were performed on QuantStudio Pro 7 (Thermo Fisher Scientific, Waltham, MA, USA) under the following thermocycling conditions: 2 min at 95 °C, 40 cycles of 10 s at 95 °C and 60s at 56 °C. A melt curve analysis was performed in increments of 0.5 °C/s from 60–95 °C.

### 4.7. Analysis of miRNome PCR Panels I + II, V5

Amplification and melting curve analysis of the 752 miRNA assays included in the miRNome PCR Panels was performed using Design & Analysis 2 (DA2) 2.6.0 software (Applied Biosystems, Thermo Fisher Scientific, Waltham, MA, USA) for each cDNA pool with the threshold set at 0.15 and automatically determined baseline. Data of miRNA assays with Cq values ≥ 37 were omitted from the analysis. Interplate calibration was performed using the Cq values of interplate calibrators, which were included in the miRNome PCR panels. Normalization and ΔCq calculation were performed using the geometric mean of Cq values of selected reference miRNA. Overall, 12 miRNAs (Table 2) with the lowest standard deviation across all pools were selected.

Selection of target miRNAs for further validation experiments with qPCR was performed based on their expression profiles (dysregulation > 2-fold) and a thorough literature overview of miRNAs involved in the processes of fibrosis. Relative miRNA fold change was calculated with the 2^−ΔΔCt^ method [44]. The expression data for all miRNAs included can be found in Supplementary Table S1. Our final selection encompassed 8 target miRNA (*hsa-miR-193a-5p*, *hsa-miR-335-5p*, *hsa-miR-376c-3p*, *mmu-miR-378a-3p*, *hsa-miR-133b*, *hsa-miR-424-5p*, *hsa-miR-133a-3p* and *hsa-miR-93-5p*; Table 3) with either reported profibrotic or fibroprotective effects. For further validation, instead of the mouse transcript *mmu-miR-378a-3p*, the human transcript *hsa-miR-378a-3p*, which has an extra C base at the 3′ end, was used to enable more accurate differentiation between Inf and Fib CD in humans.

### 4.8. Validation of Target miRNA

#### 4.8.1. Reverse Transcription (RT) PCR

A miRCURY LNA RT Kit (Qiagen GmbH, Hilden, Germany) was used to synthesize cDNA. A 10 μL RT reaction contained 2 μL of total RNA (10 ng), 0.5 μL of UniSp6 spike-ins, 1 μL of 10× miRCURY RT Enzyme Mix, 2 μL of 5× miRCURY RT Reaction Buffer and 4.5 μL of RNase-free water. Each RT reaction was incubated for 60 min at 42 °C and 5 min at 95 °C. If possible, cDNA was used immediately or it was aliquoted and stored at −20 °C.

#### 4.8.2. Quantitative Real-Time PCR (qPCR)

Through miRNA expression profiling and a thorough literature overview we selected 8 target miRNAs (*hsa-miR-193a-5p*, *hsa-miR-335-5p*, *hsa-miR-376c-3p*, *hsa-miR-378a-3p*, *hsa-miR-133b*, *hsa-miR-424-5p*, *hsa-miR-133a-3p* and *hsa-miR-93-5p*; Table 4) for further validation using qPCR. To enable comparable results to our previous studies, we used the previously tested reference miRNAs *(let-7e-5p*, *SNORD38B* and *hsa-miR-484*; Table 4) [41]. qPCR reactions were prepared and performed according to the manufacturer’s instructions (Qiagen GmbH, Hilden, Germany). Briefly, each 10 μL qPCR reaction contained 3 μL of 60-fold diluted cDNA, 5 μL of 2× miRCURY SYBR^®^ Green Master Mix, 1 μL of 200× ROX Reference Dye and 1 μL of 10× miRCURY LNA miRNA PCR Assay (339306, Qiagen GmbH, Hilden, Germany). qPCR thermocycling parameters were 2 min at 95 °C and 40 cycles of 10 s at 95 °C and 60s at 56 °C. A melt curve analysis was performed in increments of 0.5 °C/s from 60–95 °C. Each qPCR reaction was performed in duplicates on QuantStudio 7 Pro (Thermo Fisher Scientific, Waltham, MA, USA).

qPCR reaction efficiency was determined using serial dilutions (10-, 30-, 90-, 270- and 810-fold) of pooled RNA samples for each cohort and each miRNA under the above-described conditions. Each qPCR reaction to determine efficiency was performed in triplicates on QuantStudio 7 Pro (Thermo Fisher Scientific, Waltham, MA, USA).

### 4.9. Statistical Analysis

All data were analyzed using the IBM SPSS Statistics for Windows, Version 27.0 (IBM Corp., Armonk, NY, USA). Relative gene expression was calculated using Cq averaged between duplicates of each sample. Using the method described by Latham et al. [45], ΔCq was calculated by subtracting the Cq of the gene/miRNA of interest from the geometric mean Cq value of reference genes. Data distribution and homogeneity were tested using the Shapiro–Wilk and Levene’s tests, respectively. For normally distributed cohorts, paired and independent 2-tailed Student’s *t*-tests were used to determine significant differences between SS and SM or between Inf_SM/SS and Fib_SM/SS, respectively. For not normally distributed cohorts, the Wilcoxon signed rank test and Mann–Whitney U test were used to determine significant differences between SS and SM or between Inf_SM/SS and Fib_SM/SS, respectively. All differences were defined as statistically significant if *p* < 0.05.

## Figures and Tables

**Table 1:** Major demographic patient data.

	Fibrostenosis (n = 20)	Inflammatory Stenosis (n = 20)
Age (St. Dev.) [years]	41.1 (15.3)	45.3 (17.7)
Disease duration (St. dev.) [years]	7.6 (8.2)	6.4 (5.9)
Sex [male–female]	11:9	10:10

**Table 2:** List of miRNA used for miRNome panel data normalization.

miRNome PCR Panel	Reference miRNA Assay
Panel I	*hsa-miR-25-3p*
Panel I	*hsa-miR-92a-3p*
Panel I	*hsa-miR-151a-3p*
Panel I	*hsa-miR-320a*
Panel I	*hsa-miR-324-3p*
Panel I	*hsa-miR-671-5p*
Panel I	*hsa-miR-181a-5p*
Panel II	*hsa-miR-663b*
Panel II	*hsa-miR-664a-3p*
Panel II	*hsa-miR-320c*
Panel II	*hsa-miR-339-3p*
Panel II	*hsa-miR-320b*

Panel I and Panel II are part of miRCURY LNA miRNome Human PCR Panel I + II.

**Table 3:** Selected target miRNA for further validation and their calculated fold-change (2^−ΔΔCt^). Legend: Fib—fibrostenosis, Inf—inflammatory stenosis, SM—submucosal layer, SS—subserosal layer.

miRNA	Inf_SM vs. Inf_SS	Fib_SM vs. Fib_SS	Inf_SM vs. Fib_SM	Inf_SS vs. Fib_SS
*hsa-miR-133a-3p*	3.04	−1.61	1.76	−2.80
*hsa-miR-133b*	3.49	−1.52	2.14	−2.47
*hsa-miR-193a-3p*	1.36	2.61	−1.41	1.36
*hsa-miR-335-5p*	−3.64	−40.49	1.84	−6.05
*hsa-miR-376c-3p*	−1.87	1.05	1.24	2.43
*hsa-miR-378a-5p*	1.31	−2.61	1.15	−2.97
*hsa-miR-424-5p*	−4.99	−2.69	1.41	2.60
*hsa-miR-93-5p*	1.39	−1.22	−1.36	−2.30

**Table 4:** List of miRCURY LNA PCR assays used for validation.

miRCURY LNA miRNA PCR Assays	Catalog Number	Assay ID
*hsa-let-7e-5p*	Q339306	YP00205711
*hsa-mir-484*	Q339306	YP00205636
*SNORD38B (hsa)*	Q339306	YP00203901
*hsa-miR-133a-3p*	Q339306	YP00204788
*hsa-miR-133b*	Q339306	YP00206058
*hsa-miR-193a-3p*	Q339306	YP00204665
*hsa-miR-335-5p*	Q339306	YP02119293
*hsa-miR-376c-3p*	Q339306	YP00204442
*hsa-miR-378a-5p*	Q339306	YP00205946
*hsa-miR-424-5p*	Q339306	YP00204736
*hsa-miR-93-5p*	Q339306	YP00204715
*UniSp6*	Q339306	YP00203954

## Data Availability

The original contributions presented in this study are included in the article/Supplementary Materials; further inquiries can be directed to the corresponding author.

## Supplementary Materials

The following supporting information can be downloaded at https://www.mdpi.com/article/10.3390/ijms25168826/s1.
